# Mechanical Properties of Hybrid Ultra-High Performance Engineered Cementitous Composites Incorporating Steel and Polyethylene Fibers

**DOI:** 10.3390/ma11081448

**Published:** 2018-08-16

**Authors:** Yingwu Zhou, Bin Xi, Kequan Yu, Lili Sui, Feng Xing

**Affiliations:** 1 Guangdong Provincial Key Laboratory of Durability for Marine Civil Engineering, Shenzhen University, Shenzhen 518060, China; ywzhou@szu.edu.cn (Y.Z.); 2170338112@email.szu.edu.cn (B.X.); suill8969@163.com (L.S.); 2 The Hong Kong Polytechnic University, Hong Kong; zjzjykq@163.com

**Keywords:** ultra-high performance engineered cementitious composites, steel fiber, polyethylene fiber, high strength, high ductility

## Abstract

This paper presents the authors’ newly developed hybrid ultra-high performance (HUHP) engineered cementitious composite (ECC) with steel (ST) and polyethylene (PE) fibers. From this point on it will be referred to as HUHP-ECC. The volumes of steel and PE fibers were adjusted to obtain different mechanical properties, including compressive strength, tensile, and flexural properties. We found that tensile and flexural properties, including bending strength and ductility indexes, increased with higher PE fiber amounts but reduced with the increased ST fiber volume. Notably, the compressive strength had the opposite tendency and decreased with increases in the PE volume. The ST fiber had a significantly positive effect on the compressive strength. The fluidity of HUHP-ECC improved with the increasing amount of ST fiber. The pseudo strain-hardening (PSH) values for all the HUHP-ECC mixtures were used to create an index indicating the ability of strain capacity; thus, the PSH values were calculated to explain the ductility of HUHP-ECC with different fiber volumes. Finally, the morphology of PE and ST fibers at the fracture surface was observed by an environmental scanning electron microscope (ESEM).

## 1. Introduction

The engineered cementitious composite (ECC) was designed based on the micromechanics theory [1,2,3] and featured with the strain-hardening behavior. The conventional polyvinyl alcohol fiber (PVA) ECC has a tensile strain capacity of more than 3% and a maximum tensile strength between 3 and 6 MPa, with a fiber volume fraction of no more than 2% [4,5,6]. The tensile strain capacity of ECC is about 300 to 500 times larger than that of normal concrete [1,3]. In addition, ECC exhibits multiple cracking during strain-hardening process with a micro-crack width of less than 100 μm [7], making it a highly durable material in a wide variety of environmental exposure conditions. Recently, researchers (including the authors) used ultra-high molecular weight polyethylene fiber (UHMWPE or PE for short) to develop ultra-high performance engineered cementitious composites (UHP-ECC) [8,9,10,11]. Its compressive strength, tensile strength, and tensile strain capacity can reach 120–150 MPa, 15–20 MPa, and 8%, respectively [10,11]. The UHP-ECC already has vast development prospects in all kinds of civil engineering applications. 

Nevertheless, the fiber aspect ratio of PE fiber, i.e., the fiber length to diameter (*L*_f_/*d*_f_) was always higher than 500, which imposed a significantly negative impact of fluidity of mortar and led to the uneven fiber dispersion and the instability of tensile performance of the UHP-ECC specimen [9,10,11,12,13]. The low fluidity of UHP-ECC also caused considerable difficulty in construction. Additionally, the melting point of PE fiber is only 130–150 °C, which has led to a notable reduction in the tensile performance of UHP-ECC as the temperature increases [14,15,16,17]. Therefore, how to improve the workability and fire resistance of UHP-ECC needs to be urgently addressed.

Recently, the ultra-high performance concrete (UHPC) known for its high compressive strength and high fluidity of mortar has attracted wide attention [18,19,20]. To ensure a highly dense matrix, fine powders, such as quartz flour, silica fume, and glass powder were used in the mixture and steel (ST) fibers (1–6% (by volume)) were also added to enhance the compressive performance and fracture toughness [18,19,20,21,22]. The compressive strength of UHPC is usually greater than 150 MPa, and it has excellent fire resistance and workability [21,22,23,24,25,26]. However, the steel fiber reinforced UHPC always has experiences a tension-softening property after peak stress or a low strain-hardening property with a tensile capacity of about 0.6% due to the relatively lower fiber aspect ratio of steel fibers [27,28,29,30,31] and the maximum limit in steel fiber volume, which is more than 10 times lower than that of UHP-ECC.

Therefore, some researchers have [32,33,34,35,36,37] tried to combine reinforced cementitious composite material with two or more different fibers to improve the above concerned properties by creating a hybrid (the engineered cementitious composite). Ahmed and Maalej [37] investigated the tensile mechanical behavior of ECC hybrids reinforced with PE and ST fibers. They observed that the hybrid fiber-reinforced ECC material showed a higher tensile strain capacity than the reinforced HUHP-ECC with mono ST fibers, and a significant improvement in tensile strength compared with ECC reinforced with mono PE fibers. However, the tensile strain capacity of hybrid fiber-reinforced ECC was 3–5% and the maximum tensile strength was within 5 MPa. Both parameters represent a significant increase in functionality, which motivated further investigation into the mechanical properties of UHP-ECC hybrid concrete reinforced with ST and PE fibers.

The authors developed a UHP-ECC reinforced with hybrid ST and PE fiber (HUHP-ECC) concrete with both UHP-ECC and UHPC advantages. HUHP-ECC had good workability, tensile and flexural strain-hardening performance, as well as excellent compressive strength. Based on the design criteria of ECC [38,39], different mechanical properties, including the compressive strength, tensile, and flexural properties of HUHP-ECC, were obtained by adjusting the volume fraction of ST and PE fiber. Moreover, we were able to establish the relationships of mechanical properties and fiber content. Finally, the morphology of PE, ST fiber, and matrix was observed by using environmental scanning electron microscopy (ESEM). 

## 2. Experimental Program

### 2.1. Materials and Mix Proportions

**Figure 1:**
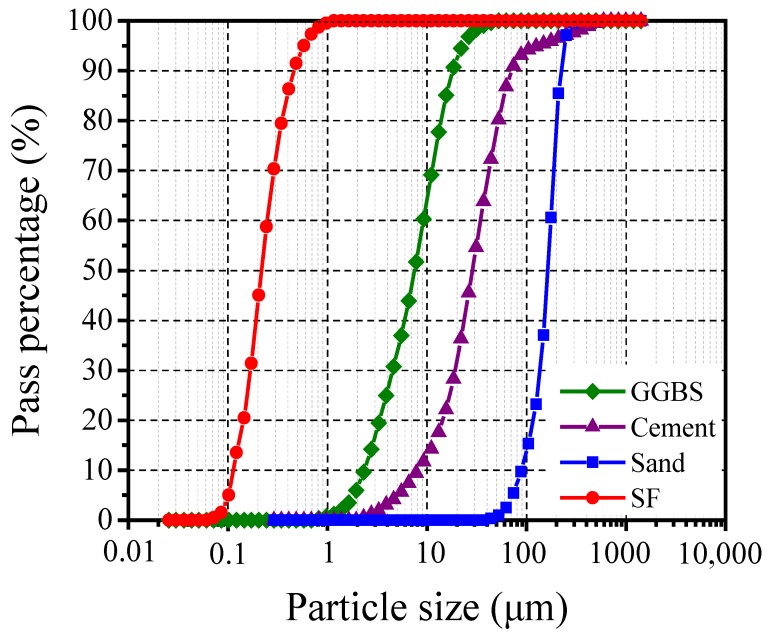
Particle size distributions of the component.

The raw materials of the HUHP-ECC binder were ordinary Portland cement 52.5R (OPC) from China Resources Cement Holdings Limited, Foshan, China, ground granulated blast furnace slag (GGBFS) and silica fume (SF). Pozzolanic materials like GGBFS and SF were incorporated to promote the formation of the secondary hydration products (calcium silicate hydrate). In addition, a polycarboxylate-based high-range water-reducing admixture (HRWRA) from Sika Co. Ltd (Sika China Group, Guangzhou, China) was used in HUHP-ECC to maintain the fluidity of the mixture at a very low water/binder ratio. Fine silica sand with a maximum grain size of 300 μm and a mean size of 100 μm was utilized as the fine aggregate. Figure 1 shows that the fine particle sizes from small to large successively are SF (0 to 1 μm), GGBFS (1 to 30 μm), OPC (2 to 300 μm) and silica sand (50 to 300 μm). The chemical compositions and the physical and mechanical properties of the cementitious materials including OPC, SF and GGBFS are listed in Table 1.

Table 2 presents the properties of steel (ST) fiber and polyethylene (PE) fiber. Both fibers have tensile strength larger than 2700 MPa. The steel fibers used in this study were straight and with a circular cross-section. The fiber aspect ratio (fiber length to diameter ratio, L_f_/d_f_) of PE is 720 and is much larger than that of steel fiber (65), which would cause tensile properties to have different effects. In addition, the different elastic moduli of the two fibers would also affect the compressive performances. The total volume fraction of steel fiber and PE fibers was kept at 2%. The mixtures were named as HUHP-ECC-A–B, where A meant the PE fiber content, and B meant the steel fiber content. A total of five mixtures were set with both PE fiber and ST fiber ranging from 2.0% to 0% with an interval of 0.5%. Take for example the HUHP-ECC-1.5-0.5, it means the HUHP-ECC specimen contains 1.5% of PE fiber and 0.5% ST fiber. Detailed mix proportions of HUHP-ECCs are listed in Table 3.

### 2.2. Mixing Procedure

All the mixtures were made in a vertical axis and speed adjustable mixer. First, dry powders and aggregates were mixed for about 2 min. The mixing speed was set at 140 rpm for 3 min after the addition of water (with HRWRA) and then set at 420 rpm for another 2 min. ST fibers were first added; then, the PE fibers were added in two batches to obtain better fiber dispersion. Before casting, the spread fluidity value of all the mixtures was measured according to the standard GBT 2419-2005 [40]. The specimens were kept in the mold for 24 h at room temperature. After demolding, all the specimens were cured in a curing room with a temperature of 23 ± 3 °C and humidity of 95% for 28 days.

### 2.3. Test Specimens and Test Setups

The experimental investigations, including the four-point bending tests and the uniaxial tension/compression tests, were implemented in the present study. In addition, the matrix fracture toughness test and the single crack tension test were conducted as well to obtain the matrix fracture energy *J*_tip_ and fiber bridging complementary energy *J*_b_′, respectively. Table 4 lists the corresponding specimen numbers for each mix proportion.

**Figure 2:**
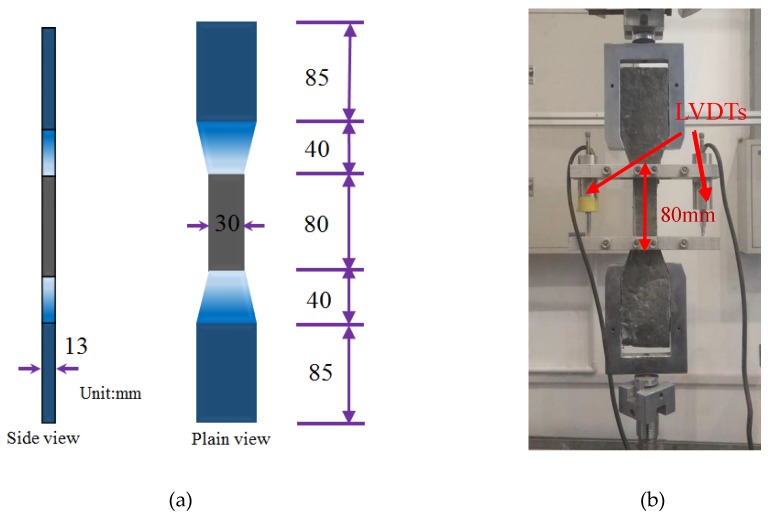
Dogbone specimen for engineered cementitious composite (ECC) tensile test. (**a**) Dimension of dog bone specimen; (**b**) Test setup.

The dogbone-shaped specimen shown in Figure 2 was cast as per the Japan Society of Civil Engineers (JSCE) [41] to carry out a uniaxial tension test. All the specimens were loaded with the MTS Landmark electro-hydraulic servo machine under displacement control at a loading rate of 1 mm/min. To determine each specimen’s tensile stress-strain relationship, two linear variable displacement transducers (LVDTs) were fixed on each side of each dogbone specimen with a gauge length of 80 mm, as shown in Figure 2. Additionally, 6 cubes (40 × 40 × 40 mm^3^) representing each mixture were tested to obtain the compressive strength.

**Figure 3:**
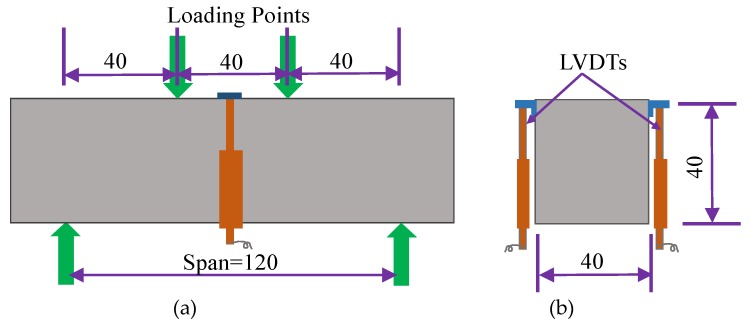
Specimen dimensions and test set up for bending test (Unit: mm). (**a**) Front elevation; (**b**) Transverse section.

To obtain the flexural properties, four-point bending tests were performed on HUHP-ECC 40 × 40 × 160 mm^3^ beams at a loading rate of 1 mm/min according to CECS (2009) [42]. The deflections at the support and midspan points were traced by two LVDTs (see Figure 3). The real mid-span deflection was then calculated by subtracting the value of the LVDTs at the mid-span point by the average displacement of the two supports. 

**Figure 4:**
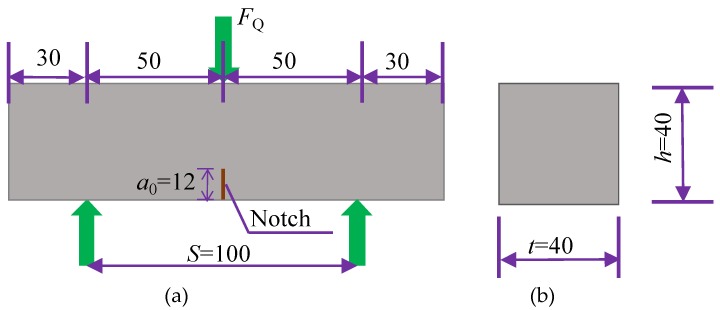
Specimen dimensions and test set up for matrix toughness test (Unit: mm). (**a**) Front elevation; (**b**) Transverse section.

To figure out the influence of matrix toughness on the tensile ductility of HUHP-ECCs, the three-point bending tests of the HUHP-ECCs matrix (without fiber) were conducted on 40 × 40 × 160 mm^3^ notched beams following the RILEM method [43] to calculate the matrix toughness of each HUHP-ECC specimen (Figure 4). Therefore, beams were cast in accordance with the mixture proportion but without adding the fibers as listed in Table 2. The initial notch/beam depth ratio was 0.3. Before testing, a notch 12 mm in depth was cut on the bottom surface at the midspan by using an electrical diamond saw. MTS Landmark electro-hydraulic servo machine was employed in the bending tests and the ultimate loads were recorded. 

**Figure 5:**
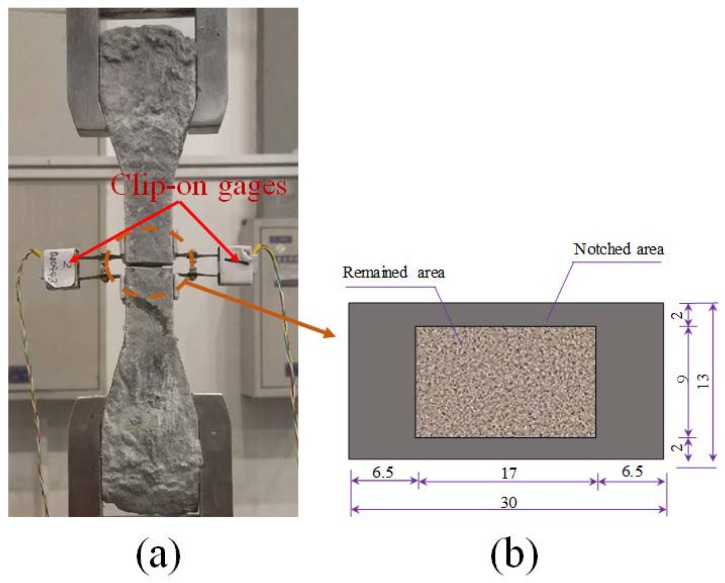
Single-crack tension test. (**a**) Instrumentation and (**b**) Notched dogbone specimen (unit: mm).

The single-crack tensile tests were carried out to obtain the bridging stress (*σ*)-crack opening (*δ*) relation *(σ-δ* curve) of HUHP-ECCs. Dogbone specimens were notched on four faces to facilitate the formation of a single crack (see Figure 5). An ultra-thin saw blade (0.4 mm in thickness) was used to cut a notch with a width less than 0.6 mm. Figure 5b shows the geometric dimension of the notched cross section of the single-crack specimen. To keep an extra crack from occurring inside or outside the notch, the adopted dimensions allowed a 61% reduction of the cross-section’s initial value. Ideally, a single crack should occur at the notch when the tensile load is applied. Two clip-on gauges with a uniform gauge length of 5 mm measured the crack opening in the test as shown in Figure 5a. 

## 3. Results and Discussion.

### 3.1. Tests of the Spread Fluidity

**Figure 6:**
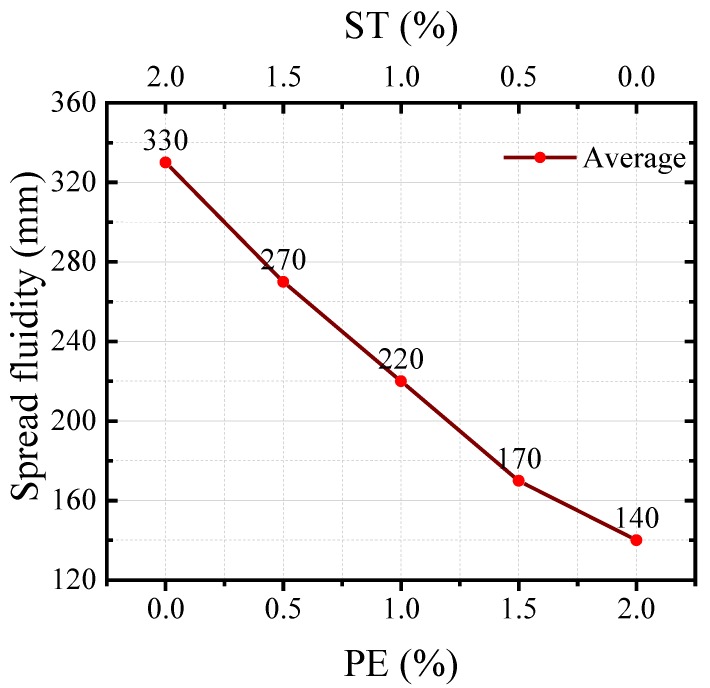
Spread fluidity of the freshly-mixed pastes of hybrid ultra-high performance (HUHP)-ECCs.

The flow behavior of concrete governs its mechanical properties. The spread fluidity value is used to evaluate the flow behavior of concrete according to the standard GBT 2419-2005 [40]. Figure 6 presents the results of the spread fluidity tests for the freshly-mixed HUHP-ECCs pastes. The spread fluidity value increased significantly with the increasing amount of steel fiber because the ST fiber number and specific surface area is smaller than the PE fiber under the same volume fraction. The spread fluidity value was 140 mm for HUHP-ECC-2.0-0 with 2% PE fiber and 330 mm for HUHP-ECC-0-2.0 with 2% ST fiber. When the steel fiber volume fraction ranged from 0.5% to 1.5%, the spread fluidity value of HUHP-ECCs increased from 170 to 270 mm, which shows the introduction of ST fiber can result in a better workability.

### 3.2. Tensile Properties

#### 3.2.1. Tensile Stress-Strain of HUHP-ECCs

**Figure 7:**
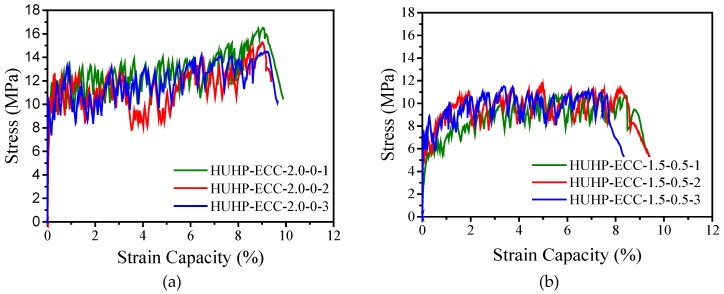

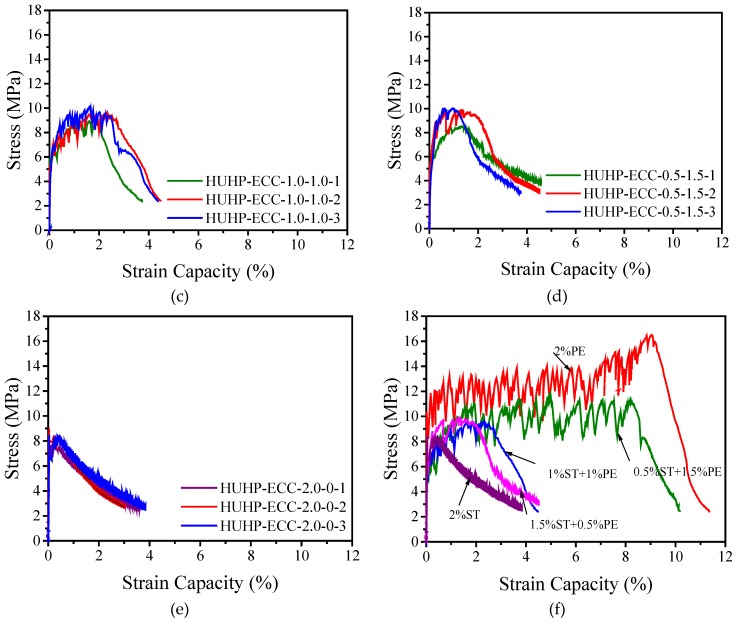
Tensile stress-strain curves of HUHP-ECCs. (**a**) HUHP-ECC-2.0-0; (**b**) HUHP-ECC-1.5-0.5; (**c**) HUHP-ECC-1.0-1.0; (**d**) HUHP-ECC-0.5-1.5; (**e**) HUHP-ECC-0-2.0; (**f**) Stress strain capacity of different volume fractions of PE and ST fibers.

Figure 7 describes the uniaxial tensile stress-strain curves of HUHP-ECCs. All the mixtures demonstrated the strain-hardening phenomenon except for the UHP-ECC-0-2.0 with 2% ST fiber, which showed a general tension-softening behavior (see Figure 7e). Figure 7f shows that PE fiber has a decisive influence on strain-hardening and tensile properties, including the tensile strength and strain capacity, both of which increased with the amount of PE fiber. The HUHP-ECC-2.0-0 with mono PE fiber reached the maximum tensile strength of around 15.5 MPa and a maximum tensile strain capacity of up to 9%, while the HUHP-ECC-0-2.0 with mono ST fiber showed a general strain-softening property and the tensile strength was only around 8.5 MPa. This is because the ST fiber had much lower fiber aspect ratio *L_f_*/*d_f_* (65) than that of PE fiber in the present research (750) and was long straight fiber with smooth surface which presented poor tensile strain capacity compared with that of hooked or twisted fiber due to the lower interfacial bond stress [32]. Notably, with 0.5% and 1.0% PE fiber, both the HUHP-ECC-0.5-1.5 and HUHP-ECC-1.0-1.0 showed a tensile strain of around 2% and a tensile strength of 9.9 MPa. Additionally, the HUHP-ECC-1.5-0.5 showed a prominent strain-hardening behavior with a strain capacity of 8% and tensile strength of approximately 12.4 MPa. Considering the better fluidity of these three mixtures, the hybrid UHP-ECCs provided alternatives for structural application.

#### 3.2.2. Tensile Parameters of HUHP-ECCs

**Figure 8:**
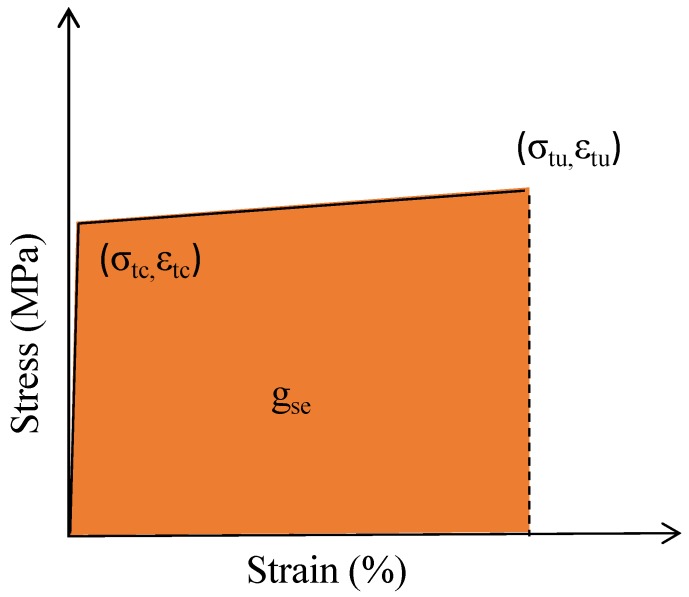
Modeled tensile stress-strain curve and critical parameters.

Figure 7 shows that the stress-strain relationship of HUHP-ECCs incorporating with different amount of ST and PE fibers feature a bilinear behavior as summarized in Figure 8, where *σ*_tc_ and *σ*_tu_ are the initial cracking strength and the peak stress, respectively. *ε*_tu_ is the strain capacity corresponding to the peak stress, and *g*_se_ is the energy absorption capacity which equates to the area enclosed with the bilinear curve.

The initial cracking stress *σ*_tc_ represents the turning point from the linear elastic portion to the strain hardening portion of the stress-strain curves. The value of the initial cracking stress *σ*_tc_ was determined from the beginning point of the strain hardening branch of the stress-strain curves. The strain energy *g*_se_ was calculated using the area underneath the ascending branch of stress-strain curves. The descending branch of the stress-stain curves during the crack localization stage was not considered in this calculation.

**Figure 9:**
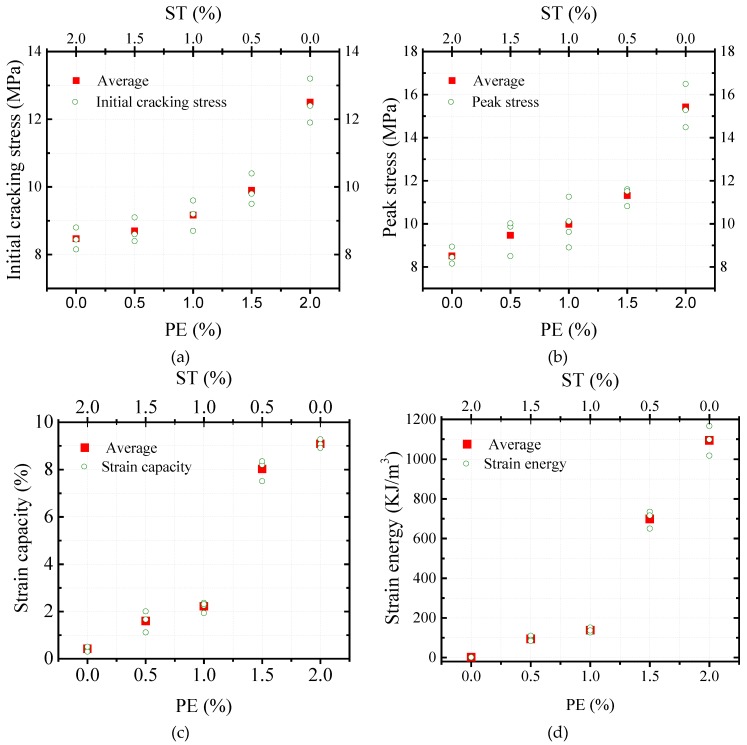
Tensile parameters of HUHP-ECCs. (**a**) Initial cracking stress; (**b**) Tensile strength; (**c**) Tensile strain capacity; (**d**) Strain energy.

Based on Figure 7 and Figure 8, the tensile parameters of HUHP-ECCs, including the initial cracking stress, tensile strength, tensile strain capacity, and strain energy, were thus obtained and their relationship with the amount of ST or PE fibers are showed in Figure 9. It could be seen that these parameters increased significantly with the amount of PE fiber due to the much higher fiber aspect ratio of the PE fiber compared to that of the steel fiber. The average initial cracking stress increased from 8.4MPa of HUHP-ECC-0-2.0 (mono ST fiber) to 12.5 MPa of HUHP-ECC-2.0-0 (mono PE fiber). The average tensile strength of HUHP-ECC-0-2.0 was 8.5 MPa and first increased steadily to 9.9 MPa of HUHP-ECC-1.0-1.0; then to 11.3 MPa of HUHP-ECC-0.5-1.5, and finally to 15.5 MPa of HUHP-ECC-2.0-0 with an increase of 182%. The average tensile strain capacity increased from 0.41% of HUHP-ECC-0-2.0 (corresponding to the onset of descending branch) to 9.1% of HUHP-ECC-2.0-0 with an increase of 23 times, which showed the decisive influence of PE fiber on the tensile strain capacity. Notably, a sudden drop occurred of strain capacity from HUHP-ECC-1.5-0.5 (8.1%) to HUHP-ECC-1.0-1.0 (2.2%), which may be attributed to the disturbance of steel fiber to the dispersion of PE fiber as the volume fraction of the PE fiber approached the critical fiber volume that could maintain the strain-hardening performance [3,4]. Additionally, the strain capacity of HUHP-ECC-1.5-0.5 and HUHP-ECC-1.0-1.0 were 2.2% and 1.6%, respectively, which was still three to four times higher than the value of UHPC [27,28]. This increased strain capacity ensures a better interaction between HUHP-ECC and steel bars at the post-yielding stage. The average strain energy shared a similar tendency with strain capacity and increased from 1.3 kJ/m^3^ of HUHP-ECC-0-2.0 to 1094.2 kJ/m^3^ of HUHP-ECC-2.0-0. The strain energies of the other three hybrid fiber reinforced UHP-ECCs are 93.6, 138.4 and 700.4 kJ/m^3^, respectively. The high strain energy would facilitate the application of HUHP-ECCs in the seismic area for energy absorption. The improvement in tensile performance of HUHP-ECCs with the increased amount of PE fiber was attributed to the larger values of the PSH index in these cases, which will be analyzed in Section 3.5.

**Figure 10:**
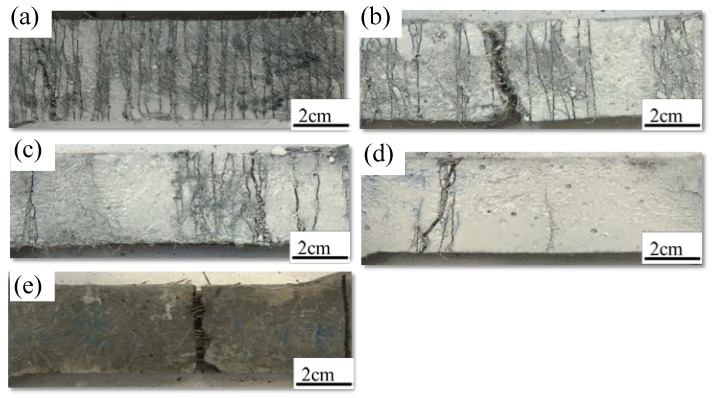
Multiple cracking behavior of different volume fractions of PE and ST fibers. (**a**) HUHP-2.0-0; (**b**) HUHP-1.5-0.5; (**c**) HUHP-1.0-1.0; (**d**) HUHP-0.5-1.5; (**e**) HUHP-0-2.0.

**Figure 11:**
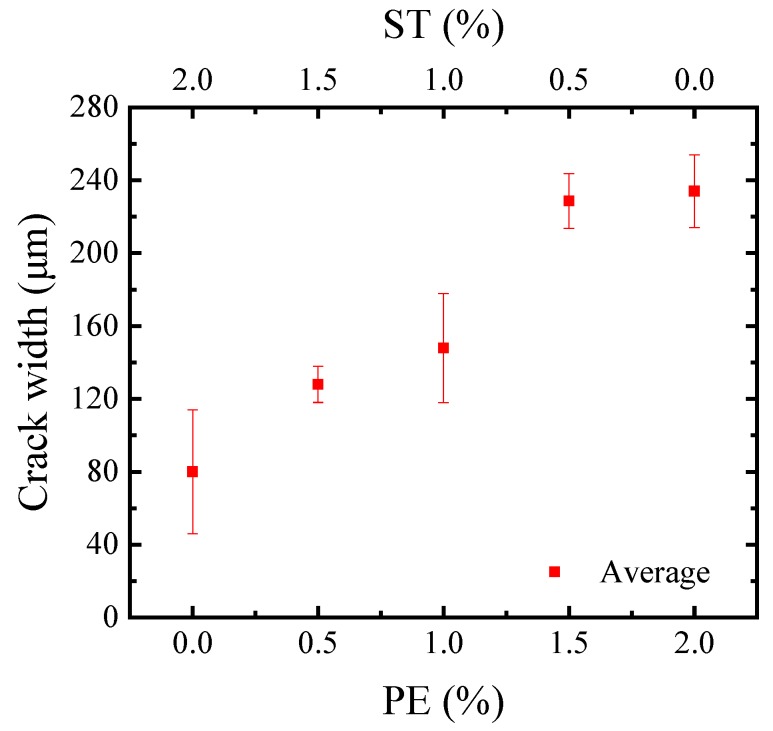
Average crack width of different volume fractions of PE and ST fibers.

Figure 10 shows the crack pattern of the HUHP-ECCs tensile specimen. With the increase of PE fiber, the HUHP-ECCs’ multi-crack phenomenon appeared obvious. HUHP-ECC-2.0-0 was saturated with micro cracks before the local failure of final crack, while HUHP-ECC-0-2.0 only triggered one main crack and then the steel fiber was pulled out from the specimen. The *L_f_*/*d_f_* of PE fiber is much larger than that of steel fiber, so it can be seen that the number of cracks is significantly determined by the PE fiber volume. Figure 11 compares the average crack width of the HUHP-ECC specimen. Notice that the increase of steel fiber reduced the crack width of the HUHP-ECC. The average crack width increased from 80 μm of HUHP-ECC-0-2.0 to 235 μm of HUHP-ECC-2.0-0. The interfacial bond stress between steel fiber and matrix is higher than that between PE fiber and matrix, so the steel fiber can be seen as having more significant effects on the restraint of crack width development, which results in the improvement of crack width control ability of HUHP-ECCs with increasing volume of steel fiber. 

### 3.3. Flexural Properties

**Figure 12:**
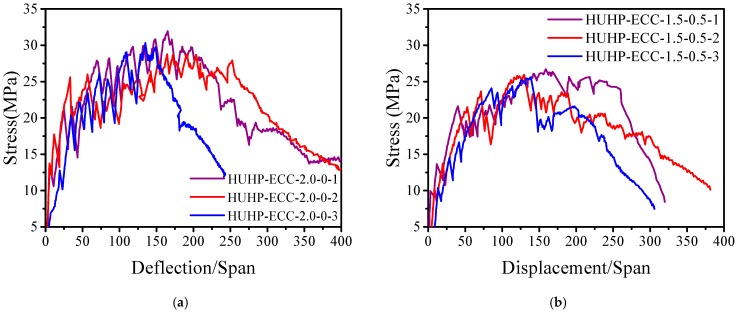

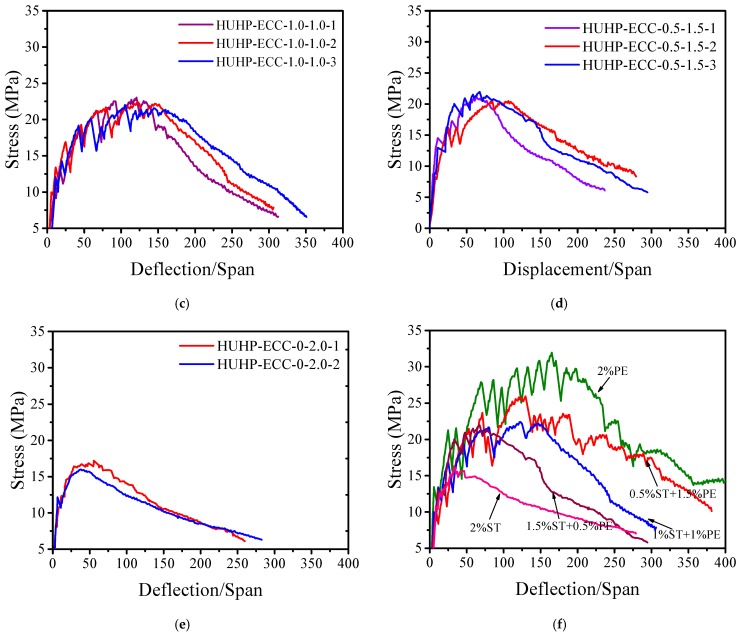
Stress versus displacement curves of HUHP-ECCs. (**a**) HUHP-ECC-2.0-0; (**b**) HUHP-ECC- 1.5-0.5; (**c**) HUHP-ECC-1.0-1.0; (**d**) HUHP-ECC-0.5-1.5; (**e**) HUHP-ECC-0-2.0; (**f**) Typical stress versus displacement curves.

Figure 12 shows the flexural stress-deflection curves of HUHP-ECCs under four-point bending. All the mixtures show flexural-hardening behavior accompanied by multiple cracks, and the deformation capacity and flexural stress increased significantly with the increase of PE fiber content as shown in Figure 12d. The average bending strength increased from 16.5 MPa at UHP-ECC-0-2.0 to 30.3 MPa at UHP-ECC-2.0-0 with an increase of 88%, and the deflection/span ratio corresponding to the peak stress increased from 1/200 of UHP-ECC-0-2.0 to 1/66 of UHP-ECC-2.0-0.

**Figure 13:**
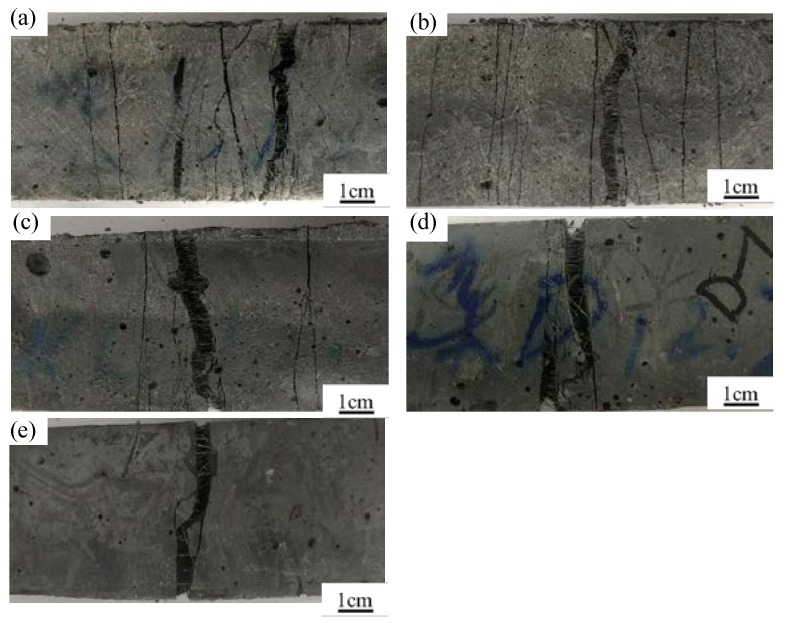
Multiple cracking of HUHP-ECCs. (**a**) HUHP-2.0-0; (**b**) HUHP-1.5-0.5; (**c**) HUHP-1.0-1.0; (**d**) HUHP-0.5-1.5; (**e**) HUHP-0-2.0.

The crack distribution at the bottom surface of the HUHP-ECC specimen after failure is shown in Figure 13. The flexural cracks normally appeared in the pure bending zone and localized at the failure stage. The crack number increased gradually with the increasing amount of PE fiber and the distribution was more uniform.

### 3.4. Compressive Properties

**Figure 14:**
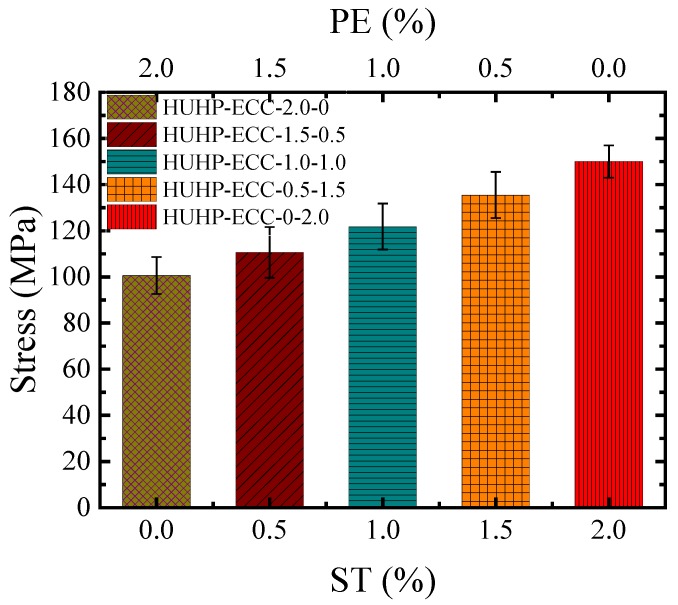
Comparison of compressive strengths of different volume fractions of PE and ST fibers.

The compressive strength of HUHP-ECC was obtained by loading the cube specimens of 40 × 40 × 40 mm^3^ up to failure at a loading rate of 2 kN/s. At least four cube specimens were used for each mixture and the average value was calculated. As shown in Figure 14, the average compressive strength decreased with the increasing amount of PE fiber. In other words, the addition of ST fiber increased the compressive strength of HUHP-ECCs. The compressive strength increased from 99.5 MPa of HUHP-ECC-2.0-0 with 0% ST fiber to 110.6 MPa of HUHP-ECC-1.5-0.5 with 0.5% ST fiber and to 135.5 MPa of HUHP-ECC-0.5-1.5 with 1.5% ST fiber. The compressive strength of HUHP-ECCs with mono 2% ST fiber showed the highest value of 150.5 MPa. The ST fiber had a significantly positive effect on the compressive strength due to the different lateral elastic moduli of these two fibers. Higher PE fiber quantities disturb the density of the matrix and induce more pores, which reduce compressive strength [44].

### 3.5. Interpretation of High Ductility in HUHP-ECC

**Figure 15:**
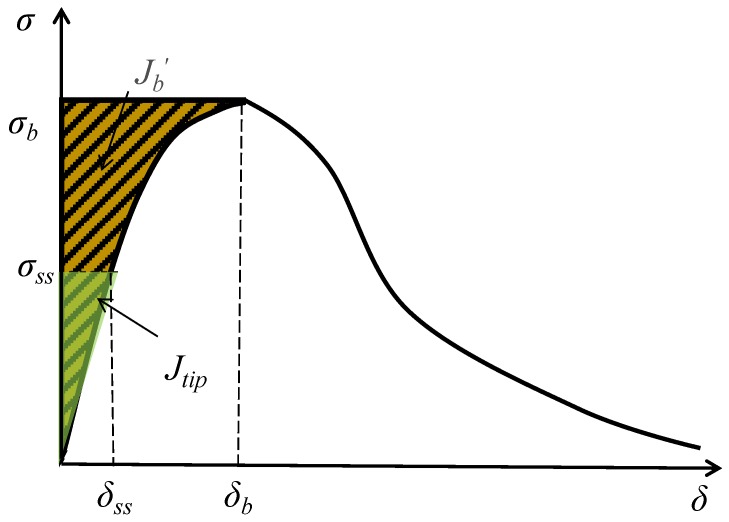
Typical σ–δ curve for strain-hardening composite.

In this study, the classic pseudo strain-hardening (PSH) performance index was also calculated to explain the ductility of HUHP-ECC. According to micromechanics [45], the pseudo-strain hardening behavior of ECC is closely related to the crack tip toughness *J*_tip_ and the fiber bridging complementary energy *J*_b_’. In summary, a relatively higher *J*_b_’ to a lower *J*_tip_ promotes flat crack propagation and leading to more cracks. Therefore, researchers calculated the ratio *J*_b_’/ *J*_tip_ which was suggested by Kanda and Li [45] as a pseudo strain-hardening (PSH) performance index for quantifying the robustness of tensile ductility in ECC (Equation (3)). Figure 15 illustrates the bridging stress *σ* versus crack opening *δ* curve, and *J*_tip_ and *J*_b_’, can be calculated by Equations (1)–(3) [2,46] ,where *E*_m_ is the elastic modulus obtained from the uniaxial tension test and the fracture toughness *K*_m_; *σ_ss_* and *δ_ss_* represent the fracture stress of matrix and the corresponding crack opening displacement, respectively.

(1) $$J_{\mathrm{tip}}\leq \sigma_{b}\delta_{b}-\int_{0}^{\sigma_{b}}\sigma (\delta )d\delta \equiv J_{b}^{’}$$

(2) $$J_{\mathrm{tip}}=K_{m}^{2}/E_{m}$$

(3) $$PSH=J_{b}^{’}/J_{\mathrm{tip}}$$

where *σ_b_* and *δ_b_* are the fiber bridging stress and the corresponding crack opening displacement, respectively. Equation (3) shows the final ratio based on (1) and (2). The fracture toughness *K*_m_ can be derived according to Equations (4)–(6) [47] based on the three-point bending test as shown in Figure 4.

(4) $$K_{m}=\frac{1.5(F_{Q}+\frac{mg}{2}\times 10^{-2})\times 10^{-3}\cdot S\cdot a_{0}^{1/2}}{th^{2}}f(\alpha )$$

where

(5) $$f(\alpha )=\frac{1.99-\alpha (1-\alpha )(2.15-3.99\alpha +2.7\alpha^{2})}{(1+2\alpha ){\left(1-\alpha \right)}^{3/2}}\;$$

and

(6) $$\alpha =\frac{a_{0}}{h}\;$$

where *m* is the mass of specimen; *F*_Q_ is the peak load of the three-point bending specimens, *g* is the gravitational acceleration; *S* is span of the three-point beam; *a*_0_ is preexisting internal flaw size; *t* and *h* are the width and height of the three-point beam. *F*_Q_, *m* and *E_m_* are obtained by the aforementioned three-point bending test and the results are listed in Table 5, as well as the calculated *K*_m_ and the crack tip toughness *J*_tip_.

**Figure 16:**
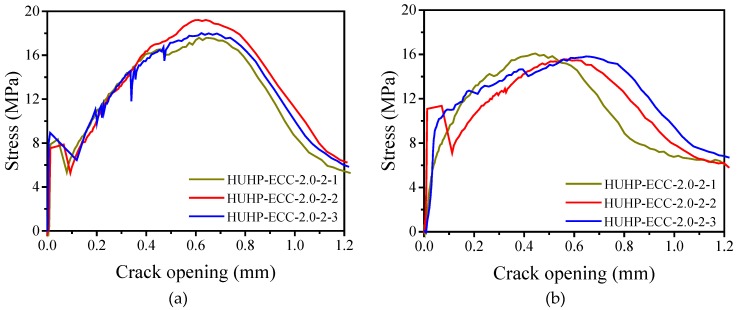

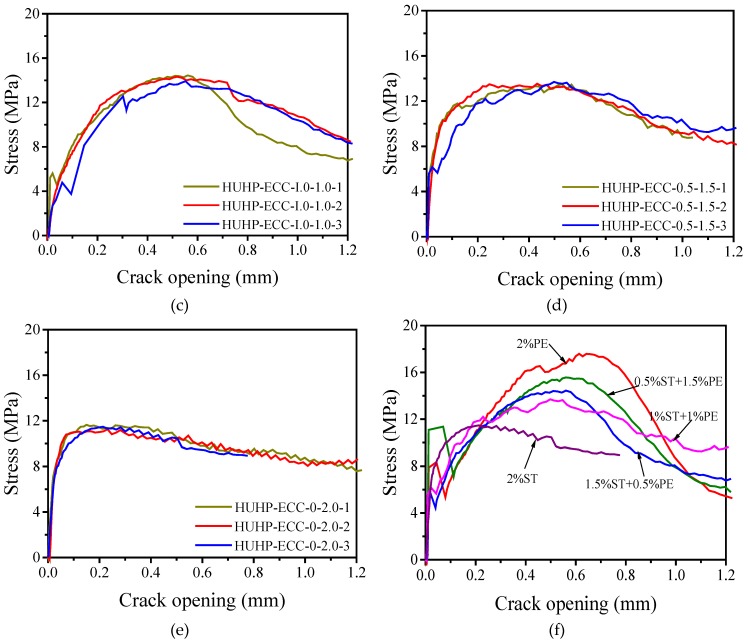
Bridging stress-crack opening displacement relationships. (**a**) Stress-crack opening curves of HUHP-ECC-2.0-0; (**b**) Stress-crack opening curves of HUHP-ECC-1.5-0.5; (**c**) Stress-crack opening curves of HUHP-ECC-1.0-1.0; (**d**) Stress-crack opening curves of HUHP-ECC-0.5-1.5; (**e**) Stress-crack opening curves of HUHP-ECC-0-2.0; (**f**) Stress-crack opening of different volume fractions of PE and ST fibers.

In Equation (1), the fiber bridging complementary energy *J*_b_′ depends on the relationship curve between the fiber bridging stress (*σ*_b_) and the crack opening displacement (*δ*_b_) of HUHP-ECCs, which can be obtained by the single crack tensile test as shown in Figure 5. The results are shown in Figure 16, from which *J*_b_’ is calculated. Figure 16d shows that both *σ*_b_ and *δ*_b_ increased with the volume fraction of PE fiber, which led a higher value of *J*_b_’ for PE fiber incorporated mixtures. The higher *L*_f_/*d*_f_ of PE fiber helped to increase the bridging stress, while the lower bond strength between the PE fiber and matrix increased the crack opening displacement. The specific values of peak bridging stress *σ*_b_ and corresponding crack opening displacement *δ*_b_ are summarized in Table 6 based on which *J*_b_’ and *PSH* are calculated and shown in Table 7.

Table 7 shows that the fiber bridging complementary energy *J*_b_′ increased significantly from 41 *J*/m^2^ to 3000 *J*/m^2^ when the PE fiber volume increased from 0% of HUPH-ECC-0-2.0 to 2% of HUPH-ECC-2.0-0. The PSH values also increased significantly with the volume fraction of PE fiber increasing from 1.8 at HUHP-ECC-2.0-0 to 112.6 at HUHP-ECC-0-2.0, which explains the increase of strain capacity of HUHP-ECCs with the higher PE fiber volume. As indicated in previous studies, the PSH should be larger than 3 to ensure a strain-hardening behavior [48]. The calculated values of PSH could well explain the tension-softening behavior of HUHP-ECC-0-2.0 and the strain-hardening phenomenon of HUHP-ECC-0.5-1.5. Therefore, high ductility and high strength HUHP-ECC can be designed by incorporating the proper proportion of ST and PE fiber volumes to yield a suitable PSH value.

### 3.6. Microstructure of HUHP-ECC

**Figure 17:**
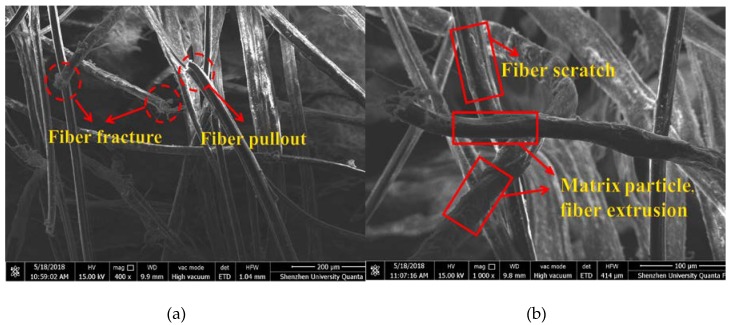

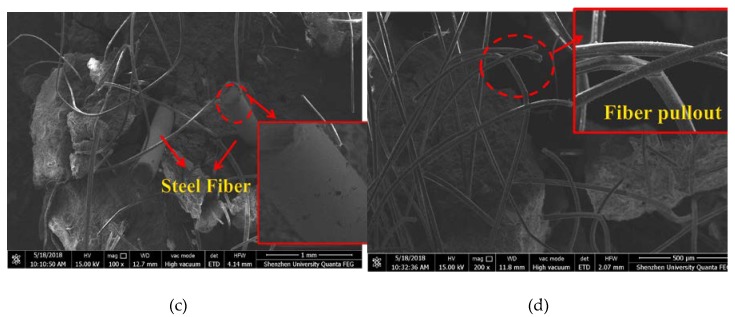
Environmental scanning electron microscopy (ESEM) images of PE and ST fiber and the fiber/matrix interface of HUHP-ECCs. (**a**) Fiber pull off surface (HUHP-ECC-2.0-0); (**b**) Fiber surface damage (HUHP-ECC-2.0-0); (**c**) Steel fiber pull out surface (HUHP-ECC-1.0-1.0); (**d**) Fiber pull out surface (HUHP-ECC-1.0-1.0).

The test results presented thus far indicate that HUHP-ECC reinforced with different amounts of PE and ST fibers shows distinct mechanical properties, which is closely related to the failure mode of the fiber. In this section, the PE and ST fiber failure modes in the fractured section after the tensile test illustrated in Figure 2 were investigated by the Quanta TM250 ESEM (FEI Company, Hillsboro, OR, USA). The morphology of tested specimens was prepared by taking small pieces from the dogbone specimens on the fractured surface. Figure 17 shows the images of PE and ST fiber failure modes in HUHP-ECC-2.0-0 specimen and HUHP-ECC-1.0-1.0 specimen Day 28. For specimen HUHP-ECC-2.0-0 reinforced with mono PE fiber, most of the PE fibers ruptured with a coinciding pull-out failure from the matrix as shown in Figure 17a. The lateral surface of the PE fiber in the HUHP-ECC-2.0-0 specimen was grooved severely on the surface or even fractured during the pull-out process as can be seen in Figure 17b. However, for specimen HUHP-ECC-1.0-1.0 reinforced with PE and ST fibers, most of the PE fibers were pulled out from the matrix with a neat end as shown in Figure 17c,d. The steel fiber surface was smooth and slightly stuck to the matrix particles; moreover, it is obvious that the diameter of ST fiber was much larger than that of the PE fiber (Figure 17c). 

## 4. Conclusions

A hybrid fiber reinforced ultra-high performance engineered cementitious composite (HUHP-ECC) was developed in the research presented herein. Different mechanical properties, which include the compressive, tensile, and flexural properties are obtained by adjusting the amounts of the fibers. The fiber combination effects on the fluidity of HUHP-ECC were also studied. The pseudo strain-hardening (PSH) values for all the HUHP-ECC mixtures were calculated to explain the ductility of HUHP-ECC. Finally, the morphology of PE and ST fiber at the fracture surface was observed by an environmental scanning electron microscope. The following conclusions could be drawn.

1. All the HUHP-ECCs demonstrated the strain-hardening phenomenon in tension except the HUHP-ECC-0-2.0 with mono ST fiber. The tensile properties, including both strength and ductility, increased with the amount of PE fiber. The tensile strain capacity of HUHP-ECC-0.5-1.5 with the 0.5% PE fiber reached 1.5%, which was much higher than the value of UHPC. The strain capacity reached 9.1% and 8.1% for HUHP-ECC-2.0-0 and HUHP-ECC-1.5-0.5, respectively. The tensile strength increased from 8.5 MPa to 15.5 MPa with an increase of 182%.
2. All the HUHP-ECCs demonstrated the strain-hardening phenomenon in flexure and the flexural strength also increased with the amount of PE fiber. While the compressive strength had the opposite tendency with the increasing PE fiber volume fraction. The addition of PE fiber imposed a negative influence on the compressive strength due to the lower lateral elastic modulus of PE fiber. Additionally, the higher quantities of PE fiber disturbed the density of the matrix and induced more pores that would reduce the compressive strength. The compressive strength of HUHP-ECC-2.0-0 and HUHP-ECC-0-2.0 were 99.5 and 150.5 MPa.
3. The fluidity of HUHP-ECC was adjustable by different combinations of ST and PE fibers. The fluidity of the HUHP-ECC increased from 140 mm with mono 2% PE fiber to 330 mm with mono 2% ST fiber. The hybrid fiber-reinforced UHP-ECCs had a spread fluidity, which ranged from 170 mm to 270 mm, with the combination of proper mechanical properties (tensile, flexural, and compressive properties) and fluidity, which may have a wider application in practical engineering.
4. The fracture toughness of the matrix and the single-crack tension test of HUHP-ECCs were conducted to obtain the PSH values to explain the tensile strain-hardening phenomenon of HUHP-ECCs. Both the peak bridging stress and the corresponding crack opening displacement increased with the volume fraction of PE fiber, which led a higher value of *J*_b_’ for PE fiber incorporated mixtures. The higher *L*_f_/*d*_f_ of PE fiber helped to increase the bridging stress, while the lower bond strength between the PE fiber and matrix increased the crack opening displacement. The morphology of the samples was studied by ESEM analysis. Without ST fiber, most of the PE fibers ruptured along with the pull-out failure from the matrix; however, with the introduction of ST fiber, the failure mode of the PE fiber mainly changed to pull out failure. The lateral surface of PE fiber in HUHP-ECC-2.0-0 specimen was grooved severely on the surface. Conversely, the steel fiber surface was smooth and slightly stuck to matrix particles, making it obvious that the diameter of ST fiber was much larger than that of the PE fiber.

## Figures and Tables

**Table 1:** Chemical properties of cement, silica fume, and ground granulated blast furnace slag (GGBFS).

**Chemical Composition (%)**	**Ingredients**	**OPC**	**GGBFS**	**SF**
	Na_2_O	0.08	0.20	0.42
	MgO	0.66	6.94	0.96
	Al_2_O_3_	4.42	12.94	0.89
	SiO_2_	19.9	39.66	92.26
	P_2_O_5_	0.10	/	/
	SO_3_	2.67	0.72	0.33
	K_2_O	0.79	1.44	1.31
	CaO	64.9	34.20	0.49
	TiO_2_	0.21	/	/
	MnO	0.10	/	/
	Fe_2_O_3_	3.00	1.58	1.97

**Table 2:** Properties of fibers.

Fiber Types	Length (mm)	Diameter (μm)	Fiber Aspect Ratio L_f_/d_f_	Modulus of Elasticity (GPa)	Fiber Strength (MPa)	Fiber Density (gm/cm^3^)
Straight Steel	13	200	65	200	2750	7.85
PE	18	25	750	116	2900	0.97

**Table 3:** Mixture proportion (kg/m^3^).

Mixture ID	Cement	SF	GGBFS	Sand	Water	Fiber volume fraction (%)		HRWRA
						PE	ST	
HUHP-ECC-2.0-0	700	230	750	500	230	2.0	0	45
HUHP-ECC-1.5-0.5	700	230	750	500	230	1.5	0.5	45
HUHP-ECC-1.0-1.0	700	230	750	500	230	1.0	1.0	45
HUHP-ECC-0.5-1.5	700	230	750	500	230	0.5	1.5	45
HUHP-ECC-0-2.0	700	230	750	500	230	0	2.0	45

**Table 4:** Numbers of replicate specimens in each test.

**Mixture ID**	HUHP-ECC-2.0-0	HUHP-ECC-1.5-0.5	HUHP-ECC-1.0-1.0	HUHP-ECC-0.5-1.5	HUHP-ECC-0-2.0
**Uniaxial Tension Test**	8	8	8	8	8
**Uniaxial Compression Test (cube)**	6	6	6	6	6
**Four-Point Flexural Test**	6	6	6	6	6
**Single Crack Tension Test**	8	8	8	8	8

**Table 5:** Fracture toughness of HUHP-ECC.

Specimen	*E_m_* (GPa)	m (kg)	*F*_Q_ (kN)	*K*_m_ (MPa m^1/2^)	*J*_tip_(*J*/m^2^)
Matrix of HUHP-ECC	35.9	0.586	1.6	0.973	26.6
S.D.	0.12	0.21	0.08	0.15	0.26

S.D. = standard deviation.

**Table 6:** Results of single-crack tension tests.

Specimen	HUHP-ECC -2.0-0		HUHP-ECC -1.5-0.5		HUHP-ECC -1.0-1.0		HUHP-ECC -0.5-1.5		HUHP-ECC -0-2.0	
	*σ*_b_ (MPa)	*δ*_b_ (mm)	*σ*_b_ (MPa)	*δ*_b_ (mm)	*σ*_b_ (MPa)	*δ*_b_ (mm)	*σ*_b_ (MPa)	*δ*_b_ (mm)	*σ*_b_ (MPa)	*δ*_b_ (mm)
Average	17.33	0.642	14.81	0.551	14.44	0.492	12.53	0.452	12.43	0.224
S.D.	0.22	0.36	0.04	0.75	1.47	2.46	0.76	1.34	2.34	0.67

S.D. = standard deviation.

**Table 7:** Results of pseudo strain-hardening (PSH).

Specimens	HUHP-ECC-2.0-0	HUHP-ECC-1.5-0.5	HUHP-ECC-1.0-1.0	HUHP-ECC-0.5-1.5	HUHP-ECC-0-2.0
***J*** **_b_** **′(*J*/m^2^)**	3000	1938	1520	500	47
**PSH**	112.6	72.7	57.1	18.8	1.8
